# *Journal of Intensive Care*: a new journal for all intensive care physicians

**DOI:** 10.1186/2052-0492-1-1

**Published:** 2013-10-23

**Authors:** Satoshi Gando

**Affiliations:** Division of Acute and Critical Care Medicine, Department of Anesthesiology and Critical Care Medicine, Hokkaido University Graduate School of Medicine, Hokkaido, Japan

I am honored to write this editorial for the launch of *Journal of Intensive Care* and sincerely welcome you to this new journal published by the Japanese Society of Intensive Care Medicine in collaboration with BioMed Central.

In the preface of the fourth edition of *Textbook of Critical Care Medicine*, the editors wrote that critical care is no longer a young specialty in medicine [1]. A decade has passed since the publication of this textbook, and intensive care medicine is now a mature and independent specialty that is taught and practiced all over the world. The area of intensive care medicine is not restricted to critical care in the intensive care unit but covers all areas of acute medicine and surgery including emergency medicine, perioperative care medicine and anesthesiology, and cardiovascular medicine. During the first decade of the twenty first century, dramatic changes and improvements have occurred in the field of intensive care medicine [2]. A large number of new theories, diagnostic techniques, and treatment strategies have been proposed, confirmed, or denied both in medical and surgical intensive care. We can also recognize great advances in the field of coronary intensive care, especially in acute coronary syndromes. In 2013, Finfer et al. [3] wrote that critical care is an all-encompassing specialty with almost limitless boundaries that involves the use of life-sustaining, high-technology medicine. They concluded that determining how to best use the available technology to our patients' benefit can only be done through high-quality research. The establishment of the intensive care field in the previous decade, as well as the large scope for expansion within this subject area, gave strength to the idea that a new, modern, internet-based electronic journal, *Journal of Intensive Care*, was needed to help further develop intensive care medicine worldwide.

*Journal of Intensive Care* is an open-access, peer-reviewed online journal that encompasses all aspects of intensive care and acute medicine. The journal aims to publish articles contributing to the development of intensive care medical science and also encourages submissions considering the different cultural aspects of intensive care practice. Open access means that all articles accepted after peer review are freely and universally accessible online via the journal website, PubMed Central [4], and other freely accessible full-text repositories. The lack of barriers to read the articles ensures that your work is disseminated to the widest possible audience immediately after publication. Therefore, *Journal of Intensive Care* has the potential to reach a much larger set of readers than any subscription-based journal, whether in print or online [5]. Another feature of open-access publishing is that the authors, not the publisher or society, hold the copyright for their work. This allows them, and anyone else, to use, reproduce, and disseminate the article, provided that it is correctly cited and no errors are introduced. It is my hope that the open-access nature of the journal will help fulfill the aim of the society and the journal to promote the exchange of ideas internationally in this and related fields. In a recent issue of *Critical Care Medicine*, it was announced that the journal has decided to decrease the number of print pages and publish some articles online only [6]. This, along with the general trend in science publishing to move to an open-access model and away from print issues and subscriptions, puts *Journal of Intensive Care* on route for great success in the future. As commitment to the journal as it begins to establish itself with researchers, the Japanese Society of Intensive Care Medicine has waived the fee which is usually associated when publishing in an open-access journal.

We believe that the reputation of *Journal of Intensive Care* depends on the articles published and so urge you to submit your manuscripts to us. We also believe in the importance of providing a fair and quick peer-review process for our authors. The journal operates a traditional closed peer-review model, and a minimum of two reviewers are consulted for each submission that proceeds to peer review. Peer review is conducted by our experienced Editorial Board members, all of whom adhere to *Journal of Intensive Care*'s editorial policy of offering a rapid, high-quality peer-review service. We continuously aim to improve the journal standing in the field by both publishing high-quality articles and providing constructive peer-review processes.

We have been editing a lot of manuscripts since we started accepting submissions a few months ago and are pleased to see submissions from not only Japan but also the Pan-Pacific countries as well as all from further afield. We have worked very hard in getting the journal ready to launch, and following this editorial, you will find the first articles to be published. We are really proud of showcasing these articles and *Journal of Intensive Care* to the world.
